# Immunophenotypic Heterogeneity and Clonal Sweep in Acute Myeloid Leukemia Revealed by Flow Cytometry: A Case Series Study

**DOI:** 10.3390/jpm16040180

**Published:** 2026-03-25

**Authors:** Angela Bertolini, Marisa Gorrese, Serena Luponio, Francesca Picone, Annapaola Campana, Francesco Verdesca, Francesca Velino, Anna Maria Sessa, Simona Caruso, Martina De Leucio, Rossella Marcucci, Anna Maria Della Corte, Pasqualina Scala, Maddalena Langella, Bianca Serio, Carmine Selleri, Valentina Giudice

**Affiliations:** 1 Hematology and Transplant Center, University Hospital “San Giovanni di Dio e Ruggi d’Aragona”, 84131 Salerno, Italy; 2 Department of Medicine and Surgery “Scuola Medica Salernitana”, University of Salerno, 84081 Baronissi, Italy

**Keywords:** acute myeloid leukemia, minimal residual disease, flow cytometry, immunophenotyping, clonal hematopoiesis

## Abstract

**Background/Objectives**: Clonal evolution is mainly defined based on the appearance or expansion of clones harboring specific somatic mutations and/or cytogenetic abnormalities, whereas few studies have investigated immunophenotypic heterogeneity assessed by flow cytometry and its relationship with disease progression. In this study, flow cytometry immunophenotyping of acute myeloid leukemia (AML) was carried out to identify phenotypic subclones based on antigen expression and to investigate clonal sweep. **Methods**: A total of 24 patients diagnosed with AML followed at the Hematology and Transplant Center of Salerno were included. Bone marrow or peripheral blood specimens were subjected to flow cytometry immunophenotyping and leukemic cell characterization. Phenotypic profiles were also compared to molecular alterations detected by next-generation sequencing. **Results**: We found that flow cytometry-defined clonal heterogeneity was more complex than molecular heterogeneity at diagnosis and disease relapse. Flow cytometry enabled the identification of small phenotypic subclones that were not detected by molecular profiling and that, in several cases, expanded over time, consistent with a phenotypic clonal sweep. The presence of small clones was associated with shorter progression-free survival and overall survival. **Conclusions**: Flow cytometric clonal heterogeneity, especially the presence of small clones (defined by antigen expression from 2 to 30%), may serve as an additional prognostic factor in AML. Immunophenotyping integrated with molecular data may improve risk stratification, enhance measurable residual disease assessment, and contribute to a more personalized disease monitoring strategy.

## 1. Introduction

Acute myeloid leukemia (AML), a clonal hematological malignancy characterized by uncontrolled proliferation of myeloid progenitor cells and a block of differentiation, is the most common blood cancer in adults in Western countries, accounting for around 3% of all cancers and 25% of leukemias, with an increasing incidence with aging [1]. Over half of AML patients are older than 65, about one-third are over 75, and these subjects generally face a poor prognosis. Standard induction therapies result in complete remission (CR) rates of only 45–55%, and fewer than 10% survive five years after aggressive treatments [2]. For elderly patients, decitabine or azacitidine—two hypomethylating agents—combined with venetoclax, a pioneering BCL2 inhibitor, have dramatically changed the prognosis of these patients, leading to improved responses and survival (CR/CR with incomplete response [CRi]: 66.4% vs. 28.3%), extending median overall survival (14.7 vs. 9.6 months), and establishing a new standard of care for this group [3]. However, high-risk AML patients, such as those with *TP53* mutations or monocytic differentiation, still experience a poor outcome [4]. Indeed, the 5-year event-free survival rate is ~50%, and one-third to one-half of patients experience disease relapse, especially those who are not eligible for hematopoietic stem cell transplantation (HSCT) [5]. Patients with persistence of measurable residual disease (MRD), also referred to as the presence of residual neoplastic cells in patients with morphological CR, show inferior clinical outcomes compared to those with sustained MRD negativity [6]. Therefore, MRD monitoring is essential for therapeutic decisions and for improving clinical outcomes of patients. Multiparametric flow cytometry monitoring is the most rapid and widely accessible platform, although sensitivity is low, with one leukemic cell detected among 10,000–100,000 normal cells [7,8]. MRD negativity by flow cytometry is defined using a threshold of <0.1% of leukemic cells on total events, as defined by the European LeukemiaNet (ELN) [9]. Although flow cytometry has several limitations, including a lack of inter-laboratory standardization based on different instruments and antibody panels used, it is still the principal method for MRD detection, as molecular biology assays are more sensitive while requiring the presence of tracking molecular alterations [10].

Clonal hematopoiesis (CH) is defined as the expansion of hematopoietic stem and progenitor cell (HSPC) clones that acquire a selective growth advantage due to acquired somatic mutations [11]. CH may represent a premalignant condition, as these mutated clones can acquire additional detrimental mutations and eventually transform into malignant conditions, such as AML [12]. Additional mutations occurring within an already expanded clone can lead to the appearance of several coexisting populations, a phenomenon known as subclonal hematopoiesis [13,14]. Aging is the main risk factor for the development of CH; indeed, although human cells have efficient mechanisms for DNA repair, somatic mutations inevitably accumulate throughout life [15]. Most of these random mutations affect non-coding regions of the genome and remain functionally silent. However, when pathogenic mutations arise together with the progressive decrease in HSPC function with age, some clones gain a selective advantage and expand; this phenomenon is known as CH of indeterminate potential (CHIP) [16,17,18]. In detail, CHIP is defined only when a somatic leukemia-associated mutation occurs in HSPCs at a variant allele frequency (VAF) ≥ 2% in the peripheral blood or bone marrow without any evidence of hematologic malignancy [19]. The most frequently involved genes are epigenetic regulators, such as *DNMT3A*, *TET2* and *ASXL1*, or those genes involved in cell signaling pathways, such as *JAK2* and *CBL*, splicing factors, including *SRSF2*, *SF3B1* and *U2AF1*, and genes responsible for DNA repair, such as *TP53* [20].

Several factors influence the likelihood that CH will progress to malignancy. For example, the presence of concomitant mutations plays a decisive role, like *DNMT3A* and *TP53* clones, which tend to expand early and slow down with aging, while mutations in splicing factors such as *SRSF2*, *PTPN11* and *U2AF1* are associated with faster expansion and a greater risk of progression to AML [21,22]. Inflammation and anticancer treatments can further modulate clonal evolution: mutations in *ASXL1* can alter the bone marrow microenvironment through inflammatory responses, creating conditions favorable for clonal expansion, while chemotherapy and radiotherapy can exert selective pressures, favoring the survival and proliferation of clones with mutations in genes involved in DNA repair [23,24,25,26].

From an evolutionary point of view, several models have been proposed to describe clonal dynamics [22]. The linear model predicts sequential progression of mutations in a single dominant clone, while the branched evolution model describes the coexistence of multiple clones derived from a common ancestor, evolving in parallel under different environmental pressures [12]. Recently, a more dynamic view of clonal evolution has emerged, as under stressful conditions, some clones can transiently expand to maintain hematopoiesis, subsequently regressing once homeostasis is restored. From this perspective, clonal evolution should not be interpreted exclusively as a precursor of malignancy, but as a dynamic adaptive process that reflects the ability of the bone marrow to respond to internal and external stimuli [27]. The transition from benign to malignant clonality is the result of an unstable balance between mutation, selection and adaptation, in which the sum of genetic and microenvironmental factors determines the outcome of the hematopoietic process [12]. The understanding of these mechanisms has profound clinical implications, as it allows the early identification of subjects at risk of AML development, the monitoring of MRD, and the advancement of targeted therapeutic strategies [28].

Clonal heterogeneity makes malignant hematologic diseases more dynamic and complex, as they are characterized by a mosaic of subclones that change over time under the pressure of the environment and therapies [16]. Therefore, the identification of CH and associated driver mutations could allow for a better understanding of the biology of hemopathies, but also for developing tools for prognostic stratification and personalized clinical surveillance, with the aim of anticipating AML transformation and guiding therapeutic strategies [29,30]. In this retrospective observational case series study, we compared molecular vs. immunophenotypic clonal heterogeneity in AML patients to investigate the potential prognostic role of immunophenotypic heterogeneity and its impact on MRD assessment. Moreover, this study aims to underline the potential value of integrating next-generation sequencing (NGS) and flow cytometry in the context of personalized medicine, capable of adapting treatment strategies based on molecular and phenotypic profiles for MRD monitoring in AML patients.

## 2. Materials and Methods

### 2.1. Patients

In this retrospective analysis, 24 AML patients (M/F, 17/7; median age at diagnosis was 74 years old; range, 68–80 years) followed at the Hematology and Transplant Center, University Hospital “San Giovanni di Dio and Ruggi d’Aragona”, Salerno, Italy, and treated with azacitidine–venetoclax, from March 2022 to October 2025, were included. The inclusion criteria were: (1) diagnosis of AML; (2) ineligibility for HSCT; (3) treatment with azacitidine–venetoclax; and (4) presence of informed consent. Classification and prognostic stratification were carried out according to 2022 World Health Organization (WHO) and 2022 ELN risk stratification criteria, respectively [31,32]. Patients’ characteristics are summarized in Table 1 and shown in detail in Supplementary Table S1.

Patients received treatments according to current international guidelines with azacytidine or decitabine and venetoclax [33]. Venetoclax dose was reduced in the case of concomitant posaconazole administration, or treatment interval was personalized according to the severity of hematological toxicity. Adverse events were assessed according to version 5.0 of the Common Terminology Criteria for Adverse Events (CTCAE). Four patients (16.7%) had previously received the following treatments as first-line therapy: one patient had received low-dose cytarabine and gilteritinib, one had received the “3+7+gemtuzumab ozogamicin” regimen, and two had received liposomal cytarabine/doxorubicin. At data cut, the percentage of disease recurrences was 37.5% (N = 9). Mortality rate was 66.7% (N = 16), mainly due to disease progression (N = 11; 45.8%). Response to treatment was assessed according to the ELN 2022 criteria [32], and the composite complete response rate (CR + CRi) was 87.5%.

### 2.2. Next-Generation Sequencing

NGS analysis was performed using SOPHIA Genetics Myeloid Solution panel (Sophia Genetics, Saint Sulpice, Switzerland), covering 30 genes (*ABL1*, *ASXL1*, *BRAF*, *CALR*, *CBL*, *CEBPA*, *CSF3R*, *DNMT3A*, *ETV6*, *EZH2*, *FLT3*, *HRAS*, *IDH1*, *IDH2*, *JAK2*, *KIT*, *KRAS*, *MPL*, *NPM1*, *NRAS*, *PTPN11*, *RUNX1*, *SETBP1*, *SF3B1*, *SRSF2*, *TET2*, *TP53*, *U2AF1*, *WT1*, *ZRSR2*) and following previously published methods [34]. Fastq files were loaded on SophiaDDM software (v4-4.6.2; Sophia Genetics) for alignment and analysis. Raw data were aligned to the human genome hg19, GRCh37, with a minimum coverage of 1000×. Non-intronic and non-synonymous variants with allele frequencies greater than 5% were considered, while intronic variants and those in the UTR region were excluded. Variant pathogenicity was determined according to COSMIC and ClinVar databases, while variants not reported in these databases were classified as pathogenic based on in silico prediction tools, such as SIFT, PolyPhen2, and FATHMM [34]. Significance of variants of uncertain significance (VUSs) was further investigated using ClinVar and VarSome databases.

### 2.3. Flow Cytometry Immunophenotyping

For immunophenotyping, 50 μL of fresh heparinized whole peripheral blood (PB) or bone marrow (BM) was incubated with specific antibodies (Table 2), as follows: for PB samples, CD3, CD4, CD5, CD7, CD8, CD11b, CD16, CD19, CD33, CD34, CD45, CD56, SmIg-kappa and SmIg-lambda; for BM specimens, CD3, CD4, CD5, CD7, CD8, CD11b, CD13, CD14, CD16, CD19, CD33, CD34, CD36, CD45, CD56, CD117, HLA-DR, SmIg-kappa and SmIg-lambda. For characterization of leukemia cells, CD45^dim/+^ leukemic cells were analyzed for the expression of CD19, CD20, CD34, CD56, CD5, CD117, CD33, CD16, CD11b, CD36, CD13, HLA-DR, CD64, CD4, CD5, CD7, CD14, CD10, CD15, CD11a, CD11c, CD45RA, CD45RO, CD61, CD66b, CD24, CD200, CD38, CD25, CD2, CD22, CD26, CD71, CD42b, TdT, and myeloperoxidase (MPO).

Briefly, blood was stained with conjugated antibodies (all from Beckman Coulter, Milan, Italy), according to the manufacturer’s instructions. After 20 min of incubation at room temperature in the dark, red blood cells were lysed by adding 2 mL of IO TEST 3 Lysing solution (stock solution 10X, Beckman Coulter). After a 10 min incubation at room temperature, samples were centrifuged at 2000 rpm for 3 min, supernatants were removed, and cell pellets were resuspended in 500 μL of sheath fluid (Beckman Coulter) and 2% of fetal bovine serum (Gibco; Waltham, MA, US). Afterwards, specimens were acquired on a Navios/EX or DxFLEX flow cytometer (Beckman Coulter), and a minimum of 500,000 events were recorded, allowing for a sensitivity of at least 0.01% [1 in 10,000 cells], in line with ELN recommendations for MRD assessment [9]. Daily quality control was performed using Flow-Check Pro Fluorospheres (Beckman Coulter) for NaviosEX or CytoFlex Daily QC Fluorospheres (Beckman Coulter) for DxFlex cytometer. External quality control was carried out by UK NEQAS for leukocyte immunophenotyping tests. The compensation was monthly verified by a Beckman Coulter specialist using the Flow-Set and the compensation kit (Beckman Coulter). Post-acquisition analysis was performed using Kaluza Analysis Flow Cytometry v2.1.1 software (Beckman Coulter).

For leukemic cell immunophenotyping, first, double cells and debris were removed using linear parameters (forward scatter [FSC]-INT vs. FSC- Time-of-Flight), and then cell populations were discriminated based on FSC vs. side scatter (SSC) area. The number of events was also assessed to further proceed with the flow cytometric analysis. Next, different cell populations were identified based on CD45 expression and on the SSC cellular complexity. In detail, lymphocytes were CD45^++^SSC^low^, monocytes were CD45^+^SSC^dim^, granulocytes were CD45^+^SSC^high^, and CD45^dim/+^SSC^dim/low^ cells were further characterized. Indeed, within CD45^dim^ cells, CD19^+^CD34^-^CD45^dim^ hematogones, CD33^+^CD117^+^CD4^+^CD56^+^ dendritic cells, CD56^+^CD16^+^CD3^+/-^CD45^+^ Natural Killer (NK) cells, CD33^+^CD11b^+^CD34^-^CD117^-^SSC^low^ basophils, CD33^++^CD13^+^CD64^++^CD11b^+^CD56^-^CD15^-^ normal monocytes, and CD33^+^CD13^+^CD64^+/dim^CD15^+^ normal granulocytes were first identified and excluded from the gate of CD45^dim^ cells. Next, leukemic CD45^dim^ cells were initially characterized by combining a second marker, such as CD34 or CD117, and then analyzed for all leukemia-associated markers. Moreover, on normal CD33^+^CD13^+^CD64^+/dim^CD15^+^ granulocytes, the maturation curve was investigated by CD16 vs. CD11b, CD13 vs. CD16, and CD64 vs. CD11b expression. In the case of CD45^dim^ lymphoid cells, a further specific multiparametric panel was applied to exclude a lymphoid leukemic clone, including SmIg κ and λ expression. Erythroid precursors were defined as CD117^+^CD33^-^CD36^+^ cells with increasing SSC. Using this gating strategy, the complete flow cytometric myelogram and leukemic cell immunophenotyping were assessed for diagnostic purposes, following international guidelines [35,36].

### 2.4. Statistical Analysis

Data were collected in spreadsheets and analyzed using GraphPad Prism (v 10.6.1; GraphPad Software, LLC, Boston, MA, US). For each marker on leukemic cells and each patient and timepoint, the percentage of positive cells on the diagnostic report was retrospectively collected in a spreadsheet. To identify possible classes of antigen expression and define clonal phenotypic heterogeneity, we divided antigen expression into four categories: negative, ranging from 0 to 1%; borderline or small clones, from 2 to 30%; positive or large clones, ranging from 31 to 89%; and positive dominant clones, from 90 to 100%.

Survival analysis was carried out by Kaplan–Meyer log-rank test. Differences between groups were studied using Chi-square, Fisher’s, Wilcoxon signed-rank, or paired or unpaired two-tailed *t*-tests. Multivariate analysis was performed by linear regression, to predict a single dependent variable using two or more independent (explanatory) variables, modeling their linear relationship to understand how these predictors influence the overall survival (OS) outcome. A *p* value < 0.05 was considered statistically significant.

## 3. Results

### 3.1. Molecular and Phenotypic CH Characterization

**Figure 1:**
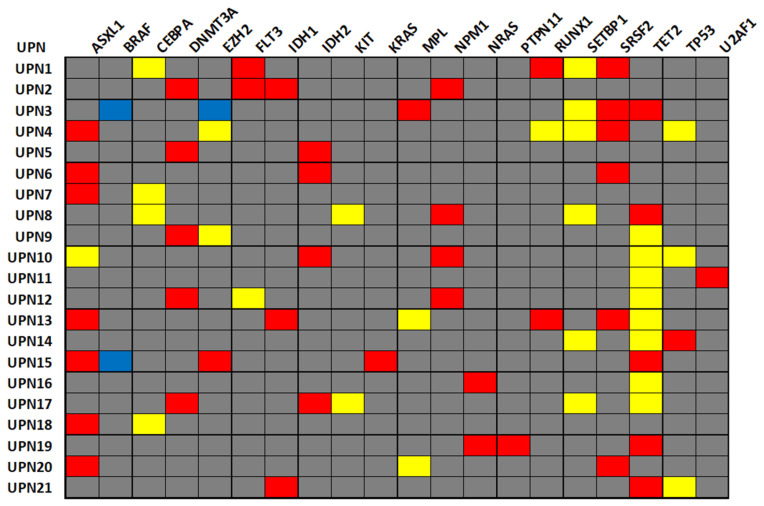
Mutational landscape of AML patients. Oncogenic alterations are colored in red, variants of uncertain significance in yellow, copy number variations in blue, and wild type in grey.

First, patients’ molecular profiles were investigated by including both pathogenic/likely pathogenic and VUS alterations (Figure 1) to identify molecular CH and recurrent alterations.

All patients showed at least one oncogenic mutation in one gene, with a median of two oncogenic alterations per patient (range, 1–4), distributed across 16 genes: *ASXL1* (9%), *SRSF2* (8%), *DNMT3A* and *TET2* (6%), *IDH2* and *NMP1* (5%), *IDH1* (4%), *FLT3*, *RUNX1* and *NRAS* (3%), and *EZH2*, *KRAS*, *MPL*, *PTPN11*, *TP53* and *U2AF1* (1%). For VUS distribution, 16 patients (67%) had at least one VUS on an AML-related gene (median 1; range, 1–4), distributed over 10 genes: *TET2* (10%), *SETBP1* (8%), *CEBPA* (5%), *TP53* (4%), *EZH2* and *KIT* (3%), and *ASXL1*, *FLT3* and *RUNX1* (1%). In particular, *SETBP1*, *KIT*, and *CEBPA* only presented VUSs. Copy number variations (CNVs) were rarely detected (9%) in *BRAF* and *EZH2*. In the other genes, neither oncogenic mutations, VUSs, nor CNVs were found. Therefore, by considering both oncogenic mutations and VUSs, all patients had at least one disease-driven alteration, with a median of three alterations per patient (range, 2–6). No statistically significant differences were observed in the distribution of oncogenic alterations when patients were divided according to the ELN risk category (*p* = 0.9459), while subjects with favorable risk tended to have a higher number of VUSs than those with intermediate (*p* = 0.0613) and adverse risk (*p* = 0.0626).

**Figure 2:**
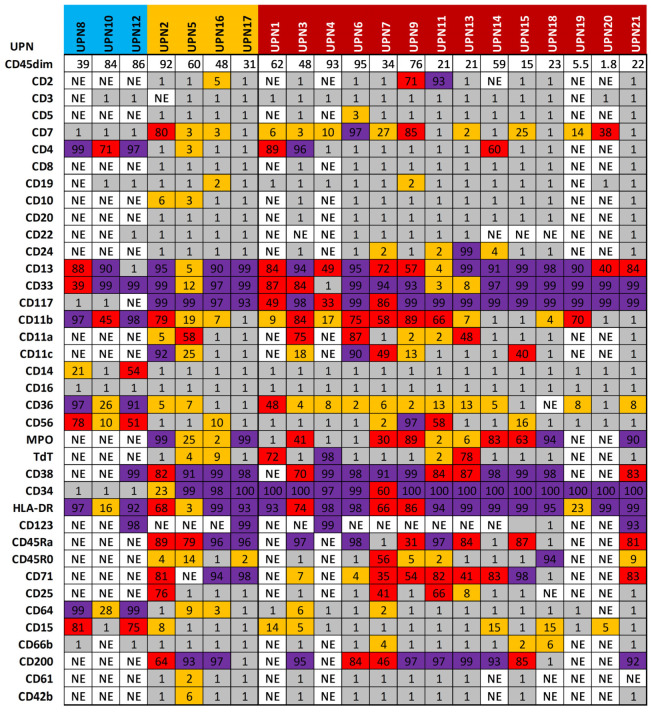
Immunophenotypic landscape of AML patients. Patients are divided according to the 2022 ELN risk category on the top (light blue, favorable risk; orange, intermediate risk; and dark red, unfavorable risk), and for each subject, antigen expression is reported in purple for the dominant clones (>90%), in red for large clones (31–89%), in orange for small clones (2–30%), and in grey in case of absent expression (≤1%). NE, not evaluated.

Subsequently, the immunophenotypic profile was evaluated in 21 out of 24 patients enrolled for whom both molecular and immunophenotypic analyses were present at diagnosis (Figure 2). We divided antigen expression into four categories: negative, ranging from 0 to 1%; borderline or small clones, from 2 to 30%; positive or large clones, ranging from 31 to 89%; and positive dominant clones, from 90 to 100%. According to this classification, in addition to the dominant clone, all AML patients had at least one subclone (median 6; range, 1–17). In particular, 18 out of 21 patients (86%) for whom gene sequencing was also available at diagnosis had at least one large clone together with the dominant one (median 3, range 1–11), and all subjects displayed at least one small subclone (median 3, range 1–15).

### 3.2. Molecular vs. Phenotypic CH

Subjects with favorable risk had a median number of large clones of three (range 2–4) and small clones of one (range 0–4), while subjects with intermediate risk had a median number of large clones of one (range, 0–8) and small clones of seven (range 1–15). Patients with adverse risk had a median number of diagnosed large clones of four (range 0–11) and small clones of three (range 1–8). Therefore, no statistically significant differences were observed in the distribution of large clones in patients based on the 2022 ELN risk stratification (*p* = 0.5094). Conversely, patients with intermediate risk tended to have a higher number of small clones than those with favorable (*p* = 0.0216) and adverse risk (*p* = 0.0448), although the number of patients per group was still very limited.

**Figure 3:**
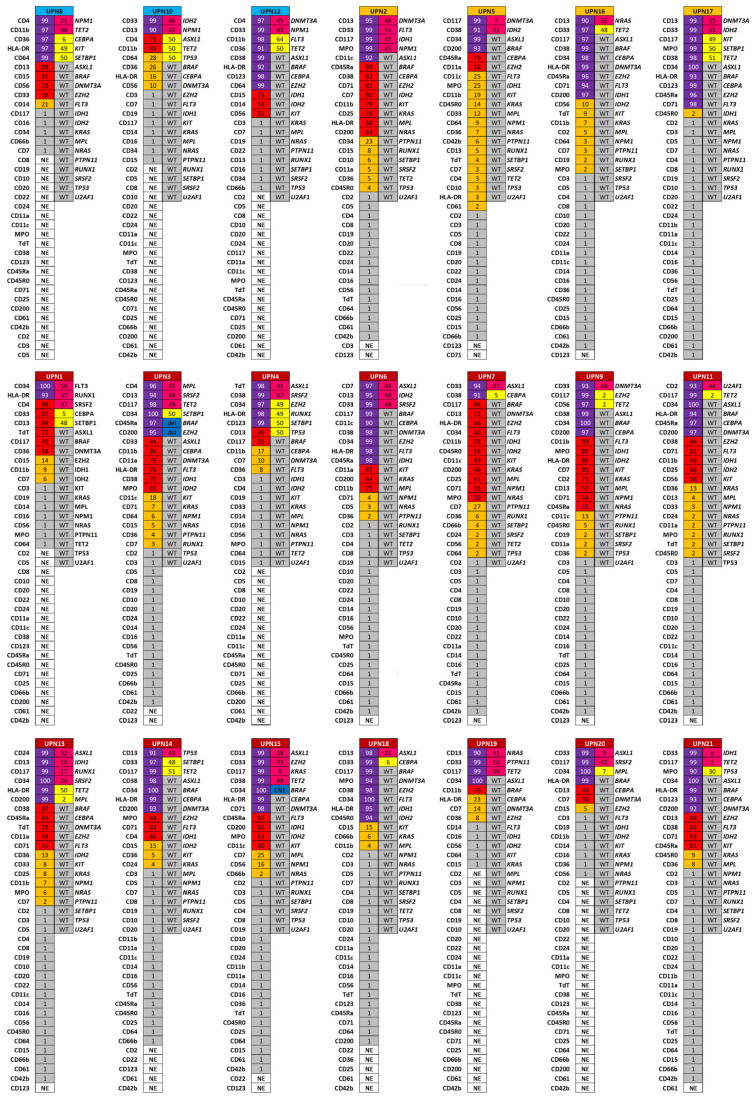
Molecular vs. phenotypic clonal heterogeneity in AML. For each patient (UPN), the left heatmap shows the percentage expression of surface markers, categorized as dominant (>90%, dark purple), large (31–89%, red), small (2–30%, orange), or negative (<1%, grey). The right heatmap shows the variant allele frequency (VAF) for mutated genes, with oncogenic mutations (light purple), variants of uncertain significance (VUSs; yellow), and copy number variations (CNVs; blue). Patients are grouped by 2022 ELN risk category (favorable, intermediate, adverse). NE, not evaluated; WT, wild type.

Next, to compare molecular and phenotypic clonal heterogeneity, molecular and immunophenotypic profiles at diagnosis were combined for each patient (Figure 3).

**Figure 4:**
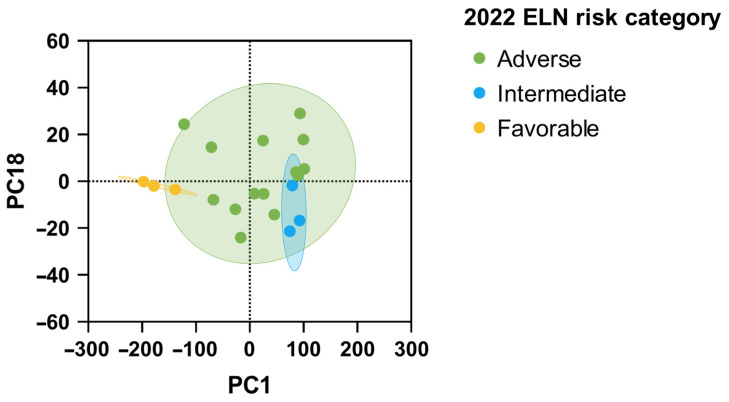
Principal component analysis by combining molecular and phenotypic data for risk stratification according to 2022 ELN categories. Clusters of patients with similar mutated genes and antigens expression are grouped based on 2022 ELN risk category (green, adverse; light blue, intermediate; and yellow, favorable).

As expected, phenotypic clonal heterogeneity was significantly more complex than molecular heterogeneity (*p* = 0.0015), with a median of six phenotypic subclones (range 1–17) vs. three molecular clones (range 2–6). Therefore, flow cytometry gave a deeper characterization of leukemic clones compared to NGS. For this reason, we sought to combine the entire mutational and immunophenotypic profiles, and AML patients were clustered by principal component analysis (Figure 4). Using this combined approach, patients with favorable risk clustered separately from those with intermediate and adverse risk, suggesting that the integration of flow cytometry and NGS may improve AML risk stratification beyond conventional molecular classification alone.

### 3.3. Prognostic Significance of Phenotypic Clonal Sweep

**Figure 5:**
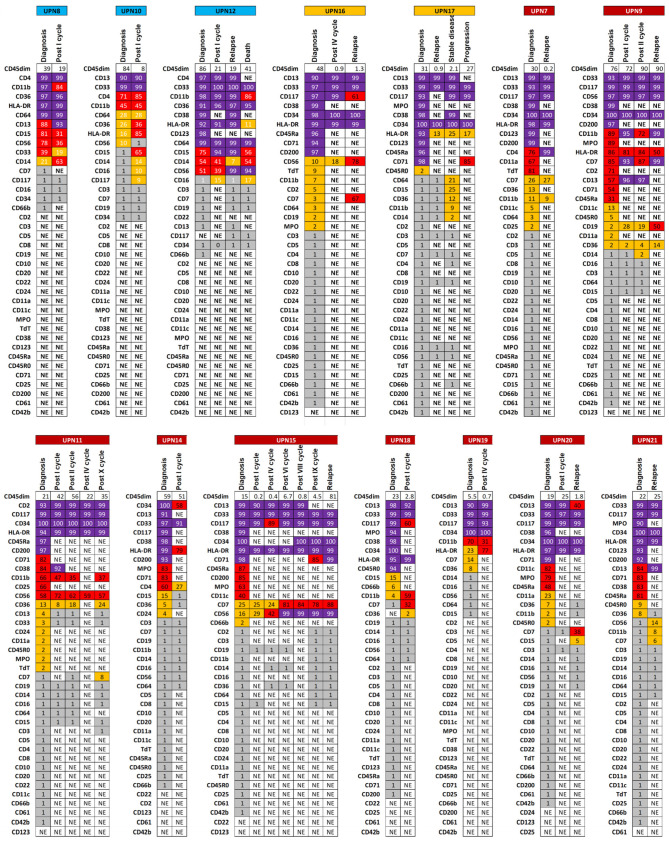
Expression of surface markers in patients with AML at diagnosis and during follow-up. For each patient (UPN), the heatmap shows the percentage expression of surface markers, categorized as dominant (>90%, dark purple), large (31–89%, red), small (2–30%, orange), or negative (<1%, grey), for each available timepoint. NE, not evaluated.

Finally, to study the clinical and prognostic significance of the presence of phenotypic subclones in AML, we investigated immunophenotypic changes during treatments and relapse in 14 subjects who had serial flow cytometry characterization (Figure 5). Qualitatively, the immunophenotypic characteristics of the dominant clone did not vary during the clinical course of the disease, while variations in the percentage of expression of small clones were observed, which, in several cases, became dominant.

**Figure 6:**
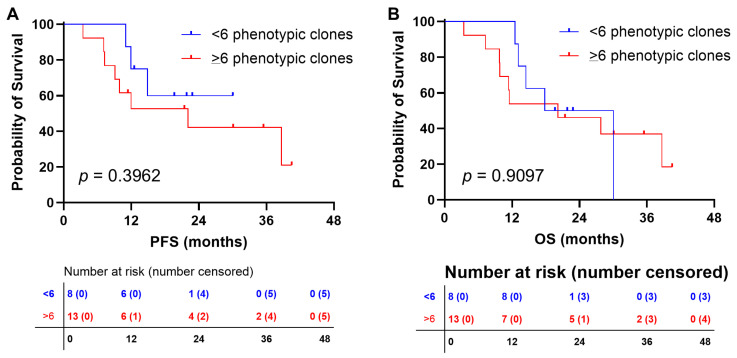
Clinical outcomes of AML patients according to clonal phenotypic heterogeneity. Patients were divided in two groups according to the number of phenotypic subclones revealed by flow cytometry: <6 clones and ≥6 clones. (**A**) Progression-free survival (PFS) and (**B**) overall survival (OS) were compared between groups.

The median number of clones was 6, and this cut-off value was used to divide patients in two groups, subjects with <6 subclones and patients with ≥6 subclones at diagnosis. Clinical outcomes were compared (Figure 6). Using this cut-off, patients with a higher clonal heterogeneity at diagnosis tended to have a shorter progression-free survival (PFS) compared to those with a lower heterogeneity (median PFS, 22.1 months vs. not reached; *p* = 0.3962), although the number of subjects per group was still limited. Moreover, among patients with adverse risk, those who had a higher number of subclones at diagnosis tended to have a shorter PFS (median PFS, not reached vs. 11.9 months, 0–3 subclones vs. ≥4 subclones at diagnosis; *p* = 0.3374), although the number of subjects was still very limited.

Preliminarily, a multivariate linear regression analysis was also conducted to identify the prognostic impact of molecular and phenotypic profiles on overall survival (OS) (Table 3). In particular, the presence of oncogenic alterations (*p* = 0.0185), VUS (*p* = 0.0231) and small phenotypic clones at diagnosis (*p* = 0.0420) emerged as a potential negative prognostic factor. In addition, a higher percentage of CD45^dim^ leukemic cells (*p* = 0.1633) and an intermediate ELN risk (*p* = 0.1408) showed a trend toward reduced overall survival. However, a larger cohort is required to confirm these data.

## 4. Discussion

AML is a complex and heterogeneous hematological malignancy with various clinical courses and worse outcomes, especially in subjects not eligible for HSCT with a 1-year relapse rate of >50% [37,38]. The 2022 version of the ELN finally introduced somatic mutations into AML risk stratification, as well as the 2022 WHO disease classification, making clinical decisions principally molecularly and clinically driven [31]. Although a molecular marker can be found in at least 80% of patients by NGS, only some of them have a clinical significance and are therapeutic changing markers, such as *FLT3* internal tandem duplication (ITD) (detectable in up to 30% of cases), guiding the use of gilteritinib, mutated *NPM1*, present in approximately 30% of cases, *CBFB*::*MYH11* in about 5% of cases, *RUNX1*::*RUNX1T1* in approximately 7% of non–acute promyelocytic leukemia cases, and *TP53* mutations, which are associated with particularly poor outcomes [39,40]. In our cohort, *FLT3*-ITD showed a relatively low representation (8%), likely because of the composition of our population in a real-world setting, with all subjects being older and unfit for intensive chemotherapy and most having MDS-related disease (66.7% of all cases). Therefore, MDS-related molecular alterations were predominant compared to molecular markers frequently detected in *de novo* diseases [41]. However, despite the molecular variability, all subjects were consistently treated with azacytidine and venetoclax, and MRD monitoring was carried out by flow cytometry immunophenotyping and circulating Wilms tumor 1 levels, thus removing a possible influence of treatment strategies on clonal sweep.

Responsiveness to induction therapies is commonly monitored by quantifying residual leukemic cells in the bone marrow, which represents an MRD [42,43]. MRD status provides valuable information for tailoring further treatments and refining prognosis. Indeed, persistent MRD is a strong predictor of poor outcomes and serves as a surrogate marker for disease-free and overall survival across various patient groups and detection methods [44,45]. Conversely, MRD negativity is associated with deeper remissions and lower relapse risk [46]. MRD can be quantified using different methodologies, including molecular techniques and multiparameter flow cytometry [47]. Current ELN guidelines recommend molecular MRD assessment only for AML with specific mutations—mutant *NPM1*, core binding factor (CBF) AML, and acute promyelocytic leukemia—using quantitative polymerase chain reaction, while flow cytometry could be used for MRD monitoring in the majority of patients, although with several assay limitations [48,49]. Flow cytometry MRD monitoring in AML faces challenges, such as lack of standardization, sample handling, staining protocols, antibody mixtures used, quantification protocols, gating strategies, and laboratory expertise [50,51]. The ELN has developed consensus recommendations to address these issues and improve consistency in MRD assessment and reporting through two main approaches: the leukemia-associated immunophenotype (LAIP) and different from normal (DfN) methods [52]. The LAIP method tracks patient-specific abnormal antigen patterns identified at diagnosis, with at least one LAIP detected in 80–95% of patients depending on the antibody panel used [9]. In contrast, the DfN approach is used to monitor aberrant immunophenotypes uncommon in healthy bone marrow during follow-up, independently of pre-treatment samples [9,53]. Both strategies require expert interpretation and some customization. The LAIP approach tends to be more sensitive but can be affected by changes in cell characteristics over time, risk of false negatives, and lower inter-laboratory agreement due to individualized panels and gating strategies. Conversely, the DfN method can be associated with false positives due to reactive blood changes after chemotherapy [43,54,55]. As each approach has distinct advantages and limitations, the ELN recommends combining both into an LAIP-based DfN approach [52]. However, neither approach considers clonal heterogeneity and subclonal hematopoiesis, resulting in a lack of monitoring of small clones potentially leading to disease relapse. In our study, we utilized intra-laboratory standardized flow cytometry panels for screening and characterization of leukemic cells at diagnosis. In particular, our screening panel allows the correct identification of B and T lymphocytes, monocytes, NK and NKT cells, HSPCs, granulocytes and maturation status, and eventually CD45^dim^ leukemic cells in the PB or BM, or aberrant expression of CD56 on mononucleated cells. Moreover, we always associated the screening panel during follow-up to add information on residual hematopoiesis. For the MRD monitoring, the combined LAIP and DfN approach was used, and some recurrent aberrant markers, such as CD36 and CD56, were often included even though they were negative at diagnosis. In addition, at disease relapse, a complete characterization of leukemic cells was carried out. This approach allowed the identification of leukemic subclones at diagnosis and their monitoring during chemotherapy and relapse. Indeed, standard MRD monitoring by LAIP and DfN approaches fails in detecting disease relapses in 35% of patients with MRD negativity after induction and consolidation therapy [56].

Tumor heterogeneity is characterized by the presence of neoplastic subclones, defined at the molecular and/or phenotypic levels, at different sites or within the same primitive lesion (spatial) or at different timepoints (temporal) [57]. The term “clonal sweep” describes a common phenomenon in solid tumors, where after induction chemotherapy, a resistant clone arises and replaces the initiator, leading to a more aggressive disease and therapy refractoriness [58]. This event is exclusively studied at the molecular level, as evolutionary trajectories are mainly studied by NGS or other molecular biology assays and are monitored by sequencing disease-associated genes [59]. For example, in multiple myeloma, plasma cells from BM and lesions have unshared mutations and chromosome alterations, such as amplification of chromosome 1, frequently found in extramedullary lesions, and those alterations are studied by multi-region whole-exome sequencing, single-cell RNA sequencing, whole-body magnetic resonance imaging or positron emission tomography [60]. These methodologies are not easily applicable to routine clinical practice, while NGS could represent a valuable additional tool to investigate spatial and temporal heterogeneity in hematological diseases [61]. However, NGS from bulk samples does not allow for precisely identifying each subclonal population, as bulk DNA sequencing captures millions of cells, giving only aggregated variant allele frequencies, although clonal deconvolution might be possible with the newest methodologies [62]. For these reasons, identification of clonal heterogeneity by flow cytometry could give a more rapid response to the clinician at the single-cell level. In addition, molecular studies by NGS only defined clonality and clonal heterogeneity based on the presence of pathogenic or likely pathogenic alterations, while VUSs are only considered a descriptive observation [63]. VUSs can be considered as variants in limbo, as they can move to either pathogenic or benign once their roles are clarified, according to published evidence [64]. We and others have previously suggested that VUSs might influence the clinical outcomes of AML and MDS patients, as, for example, the presence of multiple VUSs and/or pathogenic variants in *TET2* has been linked to better prognosis [34]. Moreover, patients with low molecular heterogeneity after HSCT show durable remission compared to those subjects who do not respond to therapy or relapse after transplantation [65]. Regardless of the specific alteration present within the hematopoietic stem cell and the pathogenicity score, the presence of VUSs might underscore a more complex phenomenon, clonal hematopoiesis, which could be the seeding soil where AML clones can arise [22]. For these reasons, phenotypic signature was combined with molecular profiling, which included both pathogenic alterations and VUSs.

The precursors of resistant disease can frequently be found as dominant subclones in focal lesions, and typically, two subclones from the primary untreated tumor are involved in the seeding of relapse cells, while multiple subclones can be seen at the same location at least once during follow-up [66]. In addition, unique subclones from different branches can be seen at distinct locations, in contrast to the classical clonal competition model, where multiple genetically distinct subclones coexist at the same location, alternating with a spatial clonal dominance pattern [59,61,67]. Although great advances have been made in clonal trajectory studies on solid tumors and hematological malignancies, the number of oncogenic mutations and driver genes remains limited compared to the extraordinary clonal heterogeneity in tumors. Indeed, although we included both oncogenic mutations and VUSs in the molecular landscapes of our AML patients, their complexity was significantly lower compared to immunophenotypic profiles, showing a median of six phenotypic subclones (range 1–17) vs. three molecular clones (range 2–6). This immunophenotypic heterogeneity could be highlighted by classifying antigen expression in four categories: negative expression (0–1%); small clones (2–30%); large clones (31–89%); and dominant clones (90–100%). This innovative approach could also have a prognostic impact, because small clones could become the drivers of disease relapse, as also shown by immunophenotypic clonal sweep in our case series. Indeed, there are no universal flow cytometry cut-off values for AML antigen positivity, as they vary by marker, purpose (diagnosis vs. MRD), and guidelines, but common thresholds include ≥20–30% for most general markers and ≥10% for MPO, with CD45 often needing ≥90% expression on blasts for identification, always interpreted with morphology and genetics [68,69]. However, there is a continuum of antigen expression from slightly negative (>2%) to borderline positivity (<20–30%), as well as positive populations, which can have a predominant antigen expression (>90%) or an intermediate positivity (30–89%). This immunophenotypic clonal heterogeneity has also been suggested in multiple myeloma, where flow cytometry could identify multiple subclones of malignant plasma cells based on differential antigen expression [70].

Our study has several limitations: (i) the small number of subjects due to the case series nature of our study, which could increase the risk of type II errors (false negatives), especially when clinically heterogenous diseases are investigated. Indeed, the wide biological variability due to patient-specific genomic and phenotypic signatures augments type II errors if subcategory sample size is not adequate; therefore, we could not exclude additional statistically significant differences between investigated groups, especially in AML entities (e.g., *TP53* mutated diseases vs. wild type, or favorable vs. adverse-risk AML). (ii) The lack of sequential immunophenotyping for the entire cohort for a comparison before and after therapies. (iii) The lack of *a priori* power analysis and a minimum detectable effect calculation.

## 5. Conclusions

NGS has improved the understanding of AML biological complexity, revealing its molecular heterogeneity, and is now routinely used in clinical practice, particularly for identifying targetable mutations, like *FLT3*-ITD, and for prognostic stratification. Nevertheless, despite these advances, optimal approaches for MRD assessment and dynamic risk evaluation remain an open challenge. In this context, multiparametric flow cytometry immunophenotyping continues to play an essential role in diagnosis and monitoring, although its prognostic potential has not yet been completely integrated into current risk models. Our LAIP-based DfN approach, incorporating both immature and mature antigen expression profiles, provides significant prognostic insights by enabling the detection of phenotypic clonal heterogeneity and clonal sweep phenomena during disease evolution. Although statistical significance was not reached due to sample size, our results suggested that the presence of small phenotypic subclones (2–30% antigen expression) at diagnosis may represent a potential risk factor for disease relapse, as clonal sweep might arise from small clones, which could be resistant to therapies or quiescent. Moreover, the combined use of NGS and flow cytometry allowed a more personalized clinical approach, risk stratification, and better MRD evaluation than either technique alone. While NGS captures a restricted set of genomic alterations, flow cytometry reveals phenotypic diversity reflecting ongoing dynamics of leukemic evolution. The absence of this heterogeneity may identify patients with more stable disease biology and potentially longer PFS. Our findings support the concept that integrated molecular and immunophenotypic profiling should be incorporated into future AML risk stratifications. This approach may improve MRD evaluation, together with the international approved ELN approach, facilitate early identification of patients at higher risk of relapse, and ultimately enable more adaptive and personalized therapeutic strategies in AML. However, further validation in larger prospective studies is needed.

## Figures and Tables

**Table 1:** Clinical characteristics.

Characteristics	N = 24
Median age, years (range)	74 (68–80)
M/F	17/7
Diagnosis, *n* (%)	
AML *de novo*	7 (29.2)
AML therapy related	1 (4.1)
AML MDS related	16 (66.7)
2022 ELN category, *n* (%)	
Favorable	4 (16.7)
Intermediate	5 (20.8)
Adverse	15 (62.5)
Median comorbidity, *n* (range)	5 (0–6)
Karyotype abnormalities, *n* (%)	
Normal	8 (33.3)
del(5q)	2 (8.3)
Single abnormality	6 (25)
Complex	2 (8.3)
Not done/failed	6 (25%)
Previous therapy, *n* (%)	4 (16.7)
Relapse, *n* (%)	9 (37.5)
Deaths, *n* (%)	16 (66.7)
Best response, *n* (%)	
CR/CRi	21 (87.5)
PR	0
SD/PD	3 (12.5)

Abbreviations. AML, acute myeloid leukemia; MDS, myelodysplastic syndrome; ELN, European LeukemiaNet; CR, complete response; CRi, incomplete count recovery; PR, partial remission; SD, stable disease; PD, progressive disease.

**Table 2:** Flow cytometry panels.

Tube	Violet Laser 405 nm		Blue Laser 488 nm					Red Laser 638 nm		
Fluorophore	PB	KO	FITC	PE	ECD	PC5.5	PC7	APC	APC A700	APC A750
Screening	CD15	CD45	DR	CD117	CD56	CD19	CD10	CD3	CD34	CD33
	CD16	CD45	CD36	CD117	CD64	CD13	CD11b	CD33	CD34	CD14
Leukemia			CD66b	CD24	CD45	CD117	CD200	CD4	CD34	CD8
	CD38	CD45	CD11a	CD13	CD45R0	CD25	CD11c	CD2	CD34	CD45RA
	CD20		CD22	CD26	CD45	CD33	CD34	CD10		CD71
			CD61	CD42b	CD45	CD13	CD5		CD34	
Cytoplasmic					CD45	CD117	CD34			
			TdT	MPO	CD45	CD117	CD34			

Abbreviations. PB, pacific orange; KO, krome orange; FITC, fluorescein isothiocyanate; PE, phycoerythrin; PC, PE-Cyanine; APC, allophycocyanin; CD, cluster designation; MPO, myeloperoxidase.

**Table 3:** Multivariate linear regression analysis (dependent variable = overall survival).

Variable	Estimate	95% CI (Profile Likelihood)	|t|	*p* Value
Intercept	−14.63	−42.40 to 13.14	1.246	0.2528
% of CD45^dim^ cells	−0.1159	−0.2918 to 0.06005	1.558	0.1633
WT1 expression	0.00013	−0.0005784 to 0.0008336	0.4273	0.6820
Favorable ELN risk	4.624	−15.58 to 24.82	0.5413	0.6051
Intermediate ELN risk	−9.501	−23.03 to 4.029	1.660	0.1408
Oncogenic mutations	8.708	1.967 to 15.45	3.054	0.0185
VUS	7.104	1.304 to 12.90	2.896	0.0231
Small clones	2.842	0.1368 to 5.548	2.484	0.0420

Abbreviations. CI, confidential interval; WT1, Wilm’s tumor 1; ELN, European LeukemiaNet; VUS, variant of uncertain significance.

## Data Availability

The raw data supporting the conclusions of this article will be made available by the authors on request.

## Supplementary Materials

The following supporting information can be downloaded at: https://www.mdpi.com/article/10.3390/jpm16040180/s1, Table S1: Patients’ characteristics.
