# Supplementary material for: Immunophenotypic Heterogeneity and Clonal Sweep in Acute Myeloid Leukemia Revealed by Flow Cytometry: A Case Series Study
Source: J Pers Med. 2026 Mar 25;16(4):180. doi: 10.3390/jpm16040180 (PMC13117096; doi:10.3390/jpm16040180)
Supplement: Supplementary file 1 [file jpm-16-00180-s001.zip › jpm-4104685-supplementary.pdf]

Table S1. Patients' characteristics.

Patient	Age	Sex	Diagnosis	ELN risk	Mutated genes	Karyotype	Therapies	Status at last follow-up
UPN1	69	F	AML-MR	Adverse	*RUNX1, SRSF2*	46,XX	Aza-ven	CR - Alive
UPN2	76	F	AML *de novo*	Intermediate	*NPM1, FLT3, DNMT3A, IDH1*	46,XX	I line: Ara-C + gilteritinib<br>II line: aza-ven	CR - Alive
UPN3	76	M	AML post MPN	Adverse	*MPL, TET2, SRSF2*	48,XY, +21, +21[15]	Aza-ven	PD – Dead
UPN4	77	M	AML-MR	Adverse	*ASXL1, SRSF2*	45, -y	Aza-ven	Second neoplasia – Dead
UPN5	73	M	AML-MR	Intermediate	*DNMT3A, IDH2*	46,XY	I line: Vixeos®<br>II line: aza-ven	PD – Dead
UPN6	76	M	AML-MR	Adverse	*IDH1, ASXL1, RUNX1*	46,XY,t(8.19)(q24;q13.1)	Aza-ven	PD – Dead
UPN7	80	M	AML-MR	Adverse	*ASXL1, EZH2, RUNX1, DNMT3A*	Failed	Aza-ven	ARDS – Dead
UPN8	79	F	AML *de novo*	Favorable	*NPM1, TET2*	46,XX	Aza-ven	Cerebral hemorrhage – Dead
UPN9	74	F	AML-MR	Adverse	*DNMT3A*	del(5q)	Aza-ven	PD – Dead
UPN10	78	M	AML *de novo*	Favorable	*IDH2, NPM1*	46,XY	Aza-ven	CR – Alive
UPN11	72	M	AML-MR	Adverse	*U2AF1*	46,XY,-20,+mar	I line: Vixeo®<br>II line: aza-ven	SD – Alive
UPN12	73	M	AML *de novo*	Favorable	*DNMT3A, NPM1*	Failed	Aza-ven	PD – Dead
UPN13	69	M	AML-MR	Adverse	*ASXL1, IDH1, RUNX1, SRSF2*	Not performed	Aza-ven	Sepsis – Dead
UPN14	79	M	AML-MR	Adverse	*TP53*	40,XY, del(1), -5, del(7),-11, -12, -16, -17, -18, add(19), -20 +mar	Aza-ven	PD – Dead
UPN15	68	M	AML-MR	Adverse	*ASXL1, KRAS, TET2*	46,XY, ider7 (total del(7p) and partial del(7q)	Aza-ven	PD – Dead
UPN16	72	M	AML-MR	Intermediate	*NRAS, TET2*	46,XY	Aza-ven	Heart failure – Dead
UPN17	71	M	AML *de novo*	Intermediate	*DNMT3A, IDH2*	Failed	Aza-ven	PD – Dead
UPN18	74	M	AML-MR	Adverse	*ASXL1, CEBPA*	46,XY	Aza-ven	CRi – Alive
UPN19	75	M	AML de novo	Adverse	*NRAS, TET2, PTPN11*	t(6;9)	Aza-ven	CRi – Alive

UPN20	70	M	AML-MR	Adverse	*RUNX1, TET2, SRSF2*	47,XY,+8[17]/46,XY[3]	Aza-ven	CR – Alive
UPN21	77	M	AML-MR	Adverse	*TP53, IDH1*	Del(5q)	Aza-ven	PD – Dead
UPN22	76	F	AML-MR	Intermediate	-	T(11;19)	Aza-ven	CR – Alive
UPN23	70	F	AML *de novo*	Favorable	*NPM1*	46,XX	I line: 3+7+GO<br>II line: aza-ven	PD – Dead
UPN24	69	F	AML-TR	Adverse	*ASXL1*	47,XX, +8,<br>t(9;22)(q34;q11.2),<br>i(17)(q10)[8] / 46,XX,<br>t(9;22)(q34;q11.2),<br>i(17)(q10)[7]	Aza-ven	PD – Dead

Abbreviations. UPN, unique patient number; AML, acute myeloid leukemia; MR, myelodysplastic syndrome-related; TR, therapy related; ELN, European LeukemiaNet; Aza-ven, azacytidine + venetoclax; CR, complete response; CRi, incomplete count recovery; SD, stable disease; PD, progressive disease; Ara-C, cytarabine; MPN, myeloproliferative neoplasm; ARDS, acute respiratory distress syndrome; GO, gemtuzumab ozogamycin.
